# Ion-Channel modulator TH1177 reduces glomerular injury and serum creatinine in chronic mesangial proliferative disease in rats

**DOI:** 10.1186/s12882-020-01842-5

**Published:** 2020-05-19

**Authors:** Andrea Cove-Smith, Claire C. Sharpe, Michael J. Shattock, Bruce M. Hendry

**Affiliations:** 1 Renal Sciences, Department of Inflammation Biology, King’s College London, Renal Medicine 10 Cutcombe Road, London, SE5 9RJ UK; 2 Barts Health Renal Centre, The Royal London Hospital, E1 1BB, London, UK; 3 Cardiovascular Sciences, The Rayne Institute, King’s College London, London, SE1 7EH UK

**Keywords:** Calcium channels, Fibrosis, Glomerulonephritis, Glomerulosclerosis, T-type, Rat, Thy-1 nephritis

## Abstract

**Background:**

T-type calcium channels (TTCC) are involved in mesangial cell proliferation. In acute thy-1 nephritis in the rat TTCC inhibition reduces glomerular damage and cell proliferation. This work is extended here by a study of the non-selective TTCC inhibitor TH1177 in a chronic model of proliferative glomerulonephritis (GN) including late treatment starting after the initial inflammation has resolved. The objective was to determine the effects of TH1177 in a model of chronic mesangioproliferative renal disease.

**Methods:**

Chronic GN was induced in WKY rats by unilateral nephrectomy (day − 7) followed by day 0 injection of Ox7 thy-1 mAb. Treatment with TH1177 (10–20 mg/Kg daily IP) was started on day 2 (early treatment) or on day 14 (late treatment) and compared to vehicle-treated controls until sacrifice at day 42. Glomerular disease was assessed with a damage score, fibrosis assay, cellular counts and renal function measured by serum creatinine.

**Results:**

Treatment with TH11777 was associated with reduced serum creatinine, less glomerular damage, reduced fibrosis and reduced glomerular cellularity. The results for early and late TH1177 treatments were essentially the same and differed significantly from vehicle.

**Conclusions:**

The ion-channel modulator TH1177 is capable of improving glomerular outcome in chronic rat GN even when treatment starts 14 days after initiation of the disease. These data are discussed in the context of the possible targets of TH1177 including TTCC, TRP family, Stim/Orai group and other cation channels. The work supports the use of genetic models to examine the roles of individual cation channels in progressive glomerulonephritis to further define the targets of TH1177.

## Background

T-type calcium channels (TTCC) are low voltage-activated (LVA) channels that have been shown to play a role in regulating the proliferative rate of non-electrically excitable cells including glomerular mesangial cells (MC) [1–3]. Selective knockdown of these channels in pulmonary artery myocytes [2] and in human MC [3] using siRNA inhibits proliferation. Blockade of the T-type current in these cell types induces cell cycle arrest at the G1/S checkpoint [3].

TH1177 is a small molecule that has been developed to inhibit extracellular calcium influx and has been shown to block TTCCs in addition to actions on other cation channels including STIM/Orai pathway and TRP channels [4]. This agent inhibits the proliferation of cultured human and rat MCs in vitro and is also a potent antiproliferative agent in prostate cancer cells in vitro and in vivo [3, 4]. We have reported that administration of TH1177 to rats with acute Thy1 nephritis reduces glomerular injury and glomerular cell proliferation in this short-term disease model [5]. This finding supports the hypothesis that TH1177 may be of benefit in renal diseases that involve excess mesangial cell proliferation.

Thy1 nephritis is a useful mechanistic tool for understanding the processes involved in MC injury and proliferative response in vivo*,* but it is far from analogous to the human diseases that we seek to modify, such as IgA nephropathy. Acute Thy1 nephritis is largely self-limiting with the injury resolving over 3–4 weeks [6]. More relevant is the development of chronic mesangial proliferative disease models using anti-Thy1 antibody injection after rat unilateral nephrectomy [7]. This strategy induces ongoing disease that more accurately mimics the pathological processes involved in the progression of chronic glomerular disease in humans [8].

Benidipine, a long-acting calcium channel blocker (CCB) that inhibits both T-type and L-type calcium channels, ameliorates glomerular injury and improves creatinine clearance in chronic mesangioproliferative nephritis in rats ([9]. Benidipine reduced renal injury compared to vehicle-treated animals and to a hydralazine-treated control group with equivalent BP response [9]. Therefore, it appears that combined T- and L-type channel blockade has therapeutic benefit in chronic mesangioproliferative GN, over and above that afforded by an effect on BP. This may be explained by the direct effect of benidipine on TTCCs in the glomerulus, but this was not examined directly. Furthermore, treatment was started on day 1 of the disease process and may have altered the disease induction phase.

The current study employs a model of chronic mesangial proliferative disease in WKY rats to examine the effect of TH1177 on glomerular injury. Importantly TH1177 treatment was commenced after the initiating insult in order to investigate whether this treatment can modify the course of established renal disease.

## Methods

All the experimental procedures were approved under provisions of the Animals (Scientific Procedures) Act 1986 and were performed under license number PPL 70/7022. Unilateral nephrectomy was performed under inhalation anaesthesia in Wistar rats weighing 200-250 g, 1 week prior to intravenous (iv) injection of 2.5 mg/kg Ox7 monoclonal antibody (day 0). The model was first characterised in groups of 3–4 animals sacrificed at weeks 1, 2, 4 and 6, compared with non-disease controls. For the therapeutic study, disease was induced in 25 animals and these were randomly assigned to one of 3 groups designated early treatment, late treatment and control. The sample size was estimated to give a 90% probability of detecting a 30% difference between groups in glomerulosclerosis at 6 weeks. At day 2 post Ox7 injection, treatment with daily intraperitoneal (IP) injections of TH1177 20 mg/kg (dissolved in DMSO) was commenced in 9 animals (early treatment group) while the remaining 16 animals received daily vehicle IP injections (DMSO only). Due to reduced rate of weight gain in the TH1177 treated animals, the dose of TH1177 was reduced to 10 mg/kg at day 12 (injection volumes/kg remained constant). At day 14, 8 animals designated the late treatment group were switched from receiving vehicle injections to receiving TH1177 10 mg/kg daily leaving 8 animals to receive vehicle injections throughout (placebo control group). Spontaneously-voided urine samples were collected at weekly intervals throughout the study. At day 42, all animals were sacrificed, serum collected by cardiac puncture and organs harvested for histological analysis. All analysis was performed by an observer blind to the treatment group of each animal. The experimental unit for analysis was an animal. One rat in the control group died prematurely and was not included in the analysis.

### Histological analysis

Sections of kidneys from animals with chronic Thy1 nephritis were stained using periodic acid Schiff (PAS) and picro Mallory trichrome (PMT). PAS stained sections were scored for glomerular injury in a blinded fashion. Thirty glomeruli per section were scored (10 from each pole and 10 from the middle) using the following scoring system:

0 = normal appearance.

1 = mesangial expansion and/or hypercellularity.

2 = microaneurysms, necrosis, capsular haemorrhage, matrix or cellular crescents.

3 = segmental sclerosis.

The average area of aniline blue staining in the glomeruli of sections stained with PMT was measured using NIS-Elements Basic Research Software (Nikon). This analysis was also performed blinded to treatment group.

### Periodic acid Schiff (PAS) staining

Formalin-fixed paraffin-embedded tissues were cut into 4 μm sections and mounted on plain glass slides, rehydrated with xylene and graded ethanols then immersed in distilled water. Sections were oxidized in 1% periodic acid for 15 min, rinsed thoroughly in distilled water and then incubated in Schiff’s reagent for 15 min. After a 5 min wash in running tap water, slides were counterstained lightly with Mayer’s haematoxylin (1 min, no differentiation in acid alcohol), washed again in tap water then dehydrated in graded ethanols and xylene and mounted.

### Picro Mallory Trichrome (PMT) staining

Sections were dewaxed and rehydrated then immersed in Celestine blue for 5 min. After being rinsed in distilled water, slides were immersed in Harris haematoxylin for 5 min then washed in tap water and rinsed briefly in 70% industrial methylated spirit (IMS). Sections were then covered with picro-orange for 10 min (extra solution was added at 5 min to avoid drying) and rinsed briefly in distilled water. Slides were incubated in ponceau fuschin for 2–3 min, rinsed in distilled water then differentiated in 1% PMA for 30–40 s (until red blood cells appeared yellow, fibrin was stained red and collagen appeared colourless). After a further rinse in distilled water, sections were immersed in aniline blue for 3–4 min, rinsed again in distilled water then dehydrated and mounted.

### Measurement of serum and urine creatinine

Creatinine concentration was measured using the Jaffé reaction. The initial rate of absorbance change was measured at a wavelength of 505 nm and compared to that of a known calibrant. A blank reaction was performed using sodium hydroxide alone to minimise interference from bilirubin.

### Measurement of urinary protein concentration

The urine protein concentration was measured using reagent supplied by Siemens Healthcare Diagnostics Ltd. (Camberley, Surrey, UK). The absorbance of the protein complex formed with pyrogallol red under acid conditions and in the presence of molybdate ions, was measured at 596 nm, and related to that of a calibration assay.

### Statistical analysis

Parametric variables were analysed using a two-tailed student’s t-test (for comparison of 2 groups) or one-way ANOVA (for multiple group comparisons), with Tukey’s multiple comparison test to compare between groups if significant. For non-parametric variables, the significance of the difference between 2 groups was determined by a two-tailed Mann-Whitney U test and multiple groups were compared using the Kruskal-Wallis test (with Dunn’s post test comparison if significant). Differences were considered significant if *p* < 0.05. Statistical analyses were performed using Prism software (Graph-Pad Software, San Diego, CA).

## Results

**Fig. 1:**
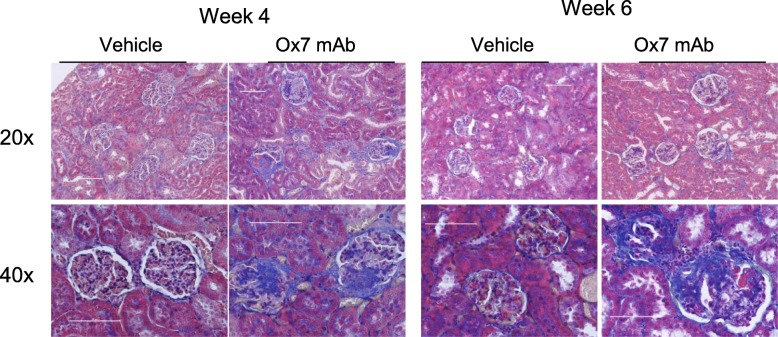
Shows PMT histology of the chronic Thy1 model at weeks 4 and 6. The disease animals show clear and progressive glomerular damage and sclerosis maximal at day 42, as shown by the blue stain. Scale bars are 100 μm

The chronic Thy1 disease model was associated with an early phase of inflammation followed by development of glomerular damage and sclerosis by day 42. Figure 1 shows the sclerosis in the model at days 28 and 42 with PMT stain showing fibrosis as blue. The model is further described by the images in Supplementary Figs. 1–3. These show progressive glomerular damage and the quantification of glomerular cell number and the patterns of Ki67 and ED1 staining as the disease progressed. By day 42 the acute inflammatory signals had resolved with no excess of Ki67 or ED1 stain compared to controls. The key endpoints chosen for the therapeutic study were the day 42 chronic glomerular injury and level of sclerosis, along with serum creatinine as an estimate of renal function.

### TH1177 reduces glomerular injury in chronic Thy1 nephritis, whether treatment is started 2 days or 2 weeks after disease initiation

**Fig. 2:**
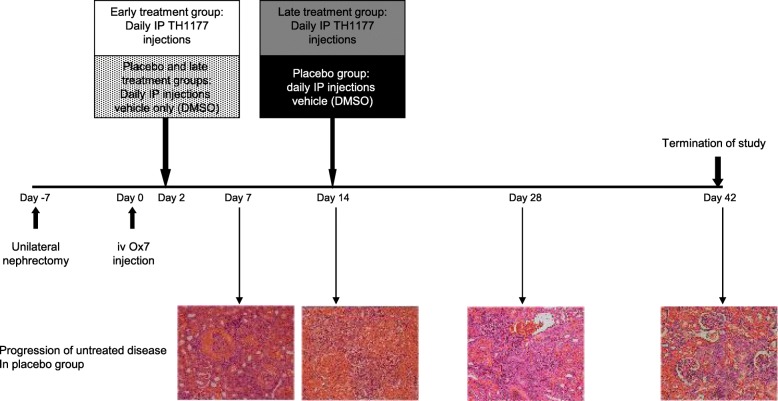
Shows the scheme of the experiment testing the effects of early or late treatment with TH1177 in rats given chronic nephritis by unilateral nephrectomy followed by Ox-7 mAb injection. Representative PAS histology sections are shown at the time points marked illustrating the disease process in vehicle-treated animals

**Fig. 3:**
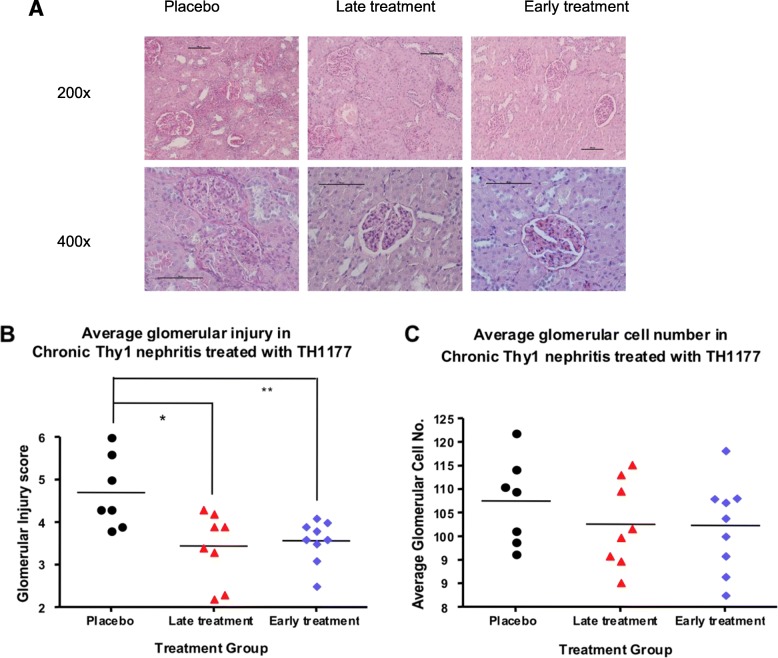
**a** shows renal sections with PAS staining for animals with chronic Thy-1 nephritis sacrificed at day 42. The 3 groups are placebo treated, late treatment with TH1177 and early treatment with TH1177. 200x and 400x magnifications are shown. Scale bars are 100 μm. **b** shows the average glomerular injury score for the 3 treatment groups. Scores from the middle and poles are added so the score is out of a maximum of 9. Glomerular injury score was reduced from an average of 4.70 ± 0.85 in the placebo group, to 3.57 ± 0.50 (*p* < 0.01) and 3.44 ± 0.81 (*p* < 0.05) in the early and late treatment groups respectively. *P* values are indicated * for < 0.05 and ** for < 0.01. **C** shows the average glomerular cell numbers for the 3 treatment groups

The scheme of the therapeutic experiment is shown in Fig. 2. All rats were subject to disease by unilateral nephrectomy and Ox7 mAb injections. They were randomly allocated to 3 groups for vehicle treatment, early TH1177 treatment or late TH1177 treatment. Placebo-treated kidneys showed evidence of glomerular hypertrophy, periglomerular fibrosis and segmental scleroses 42 days after Ox7 mAb injection (shown in the lower panels of Fig. 2). The 42 day changes were less severe in both of the TH1177-treated groups (Fig. 3a). Average glomerular injury score, performed blinded to treatment group, was significantly lower in both TH1177-treated groups than in the placebo group (Fig. 3b). Average glomerular cell number was reduced in the treatment groups (Fig 3c), but these differences did not reach significance.

### Effect of TH1177 on collagen deposition in chronic Thy1 nephritis

**Fig. 4:**
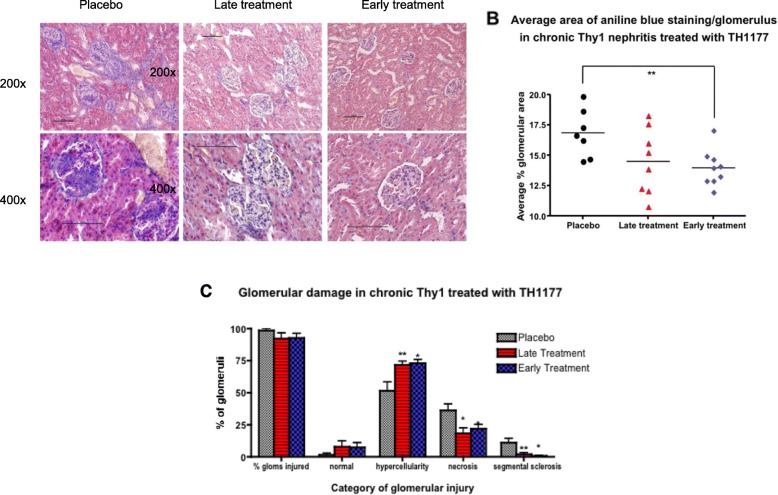
**a** shows representative examples of PMT stained renal sections from the animals with chronic thy1 nephritis for the 3 treatment groups (vehicle treated, late TH1177 treated and early TH1177 treated). The blue stain indicates matrix deposition seen maximally in the vehicle treated animals. Scale bars are 100 μm. **b** shows the area of aniline blue staining by automated image analysis in the 3 treatment groups. **c** shows the categories of glomerular damage quantified in the 3 groups. Significant differences from vehicle-treated are indicated * for *p* < 0.05 and ** for *p* < 0.01. Error bars are S.E.M.

Figure 4a shows representative images of PMT staining of sections from the 3 treatment groups. The mean percentage of aniline blue staining per glomerulus was higher in the placebo group than in the treatment groups (Fig. 4b). This difference was significant for the early treatment group (*p* = 0.034) but not for the late treatment group (*p* > 0.05) due to increased scatter. Further analysis of the distribution of glomerular injury scores revealed that there were fewer abnormal glomeruli with segmental scleroses in both treatment groups than in the placebo group (Fig. 4c). There were also significantly fewer glomeruli with cellular crescents and less evidence of cell necrosis in the TH1177-treated groups (Fig. 4c).

### Effect of TH1177 on biochemical markers of renal disease in chronic Thy1 nephritis

**Fig. 5:**
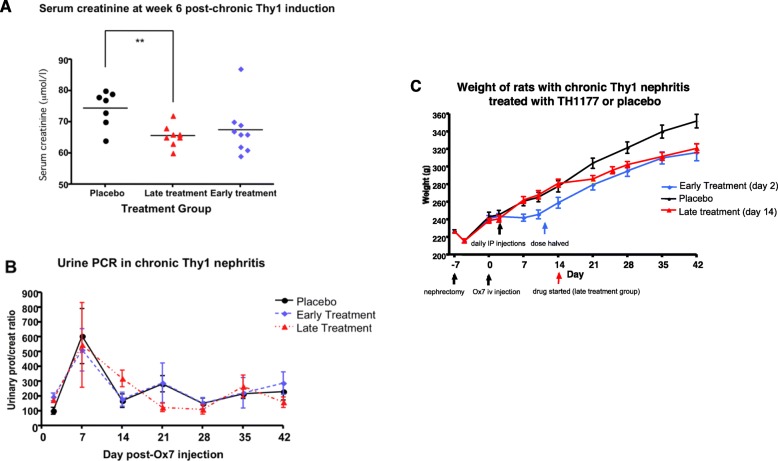
**a** shows serum creatinine at day 42 sacrifice in the animals with chronic Thy1 nephritis divided by treatment group (vehicle treated, early TH1177 treatment, late TH1177 treatment). **b** shows the time course of mean urinary PCR in the 3 treatment groups. **c** shows the time course of mean weights of animals in the 3 treatment groups. Significant differences from placebo are indicated * for *p* < 0.05 and ** for *p* < 0.01. Error bars are S.E.M.

Figure 5a shows that mean serum creatinine was similar in the two treatment groups and lower than in the placebo group. This difference from placebo was significant for the late treatment group, but not for the early treatment group due to a single outlying result. Urinary Protein Creatinine Ratio (uPCR) is shown in Fig. 5b and was not significantly different between the treatment groups. A peak in proteinuria was seen in all 3 groups at day 7 but this resolved by day 14 and remained stable in all groups up to the termination of the study.

### Rate of weight gain is reduced by IP TH1177 injections in chronic Thy1 nephritis

Initiation of TH1177 injections in this study led to a reduction in rate of weight gain compared to rats receiving vehicle injections only (Fig. 5c). The animals continued to appear well and behave normally but did not gain weight as rapidly as their vehicle-treated counterparts. Previous studies using TH1177 in rats [5] and mice [4] did not encounter this problem. At sacrifice it was noted that the rats receiving intraperitoneal TH1177 had macroscopic evidence of peritoneal sclerosis, which was not seen in any control animals. The relationship between body weight and serum creatinine at day 42 was examined for all animals in the study and no correlation was found (data shown in Supplementary Fig. 4).

## Discussion

We demonstrate here that TH1177 therapy reduced glomerular injury in rats with chronic Thy1 nephritis. This reduction is seen whether treatment is started early in the disease process (day 2 post-Ox7 injection) or delayed until after the initial inflammatory phase has abated (day 14 post-Ox7 injection). There is also a significant reduction in collagen deposition in the glomeruli in the early treatment group (as assessed by aniline blue staining) and a reduction in serum creatinine in the late treatment group. These findings support the hypothesis that TH1177 can slow the progression of kidney damage in mesangioproliferative renal disease.

In this study, treatment was started after disease induction in an attempt to address the question of whether TH1177 inhibition can modify the course of established proliferative renal disease. The time of treatment initiation was based on a pilot study analysing the time-course of this disease model in our hands. By day 14 of this model, the acute glomerular injury and interstitial inflammation seen in response to iv Ox7 mAb had largely resolved, leaving minimal residual inflammation but ongoing glomerular hypercellularity and increased mesangial matrix deposition. In untreated disease, these findings progressed up to day 42, resulting in further glomerular hypertrophy with areas of glomerulosclerosis and interstitial fibrosis. Therefore, the two treatment groups were designed to investigate the impact of TH1177 treatment on the initial proliferative response of MC (in the case of the early treatment group, day 2) and on the ongoing glomerular hypertrophy, MC proliferation and matrix accumulation, even after this process has been established (late treatment group, day 14).

In the acute Thy1 nephritis model, with TH1177 treatment initiated at the time of disease induction, there is an impact on glomerular cell proliferation, demonstrating that TH1177 can inhibit MC proliferation in vivo as well as in vitro (5). In the current chronic model, the rate of MC proliferation in the later stages is slower and this is reflected in the lower number of Ki67-positive nuclei/glomerulus seen in this disease at weeks 4 and 6 compared to the acute model (Supplementary Fig. 3 and reference [5]). There are also a number of glomeruli in the more severely diseased kidneys that have developed overt scleroses by day 42, which are hypocellular lesions with few actively proliferating cells within them. In the analysis of Fig. 4c the placebo group showed a lower % of glomeruli with hypercellularity. This was surprising as the in vitro data and acute Thy1 results have shown TH1177 to reduce proliferation [3, 5]. The current result may arise from the increased number of (hypocellular) sclerosed glomeruli in the placebo arm of the study rather than reflecting a true hyperproliferative action of TH1177.

The precise molecular target of TH1177 in this study is not established and actions on cation channels other than TTCC are entirely possible, including effects on Stim/Orai pathways and TRP channels [10], and these could be pivotal in the biological effects seen. In particular, TRPC family channels are implicated in glomerular pathology [11] and may be influenced by TH1177. Like all channel inhibitors, TH1177 is not 100% specific and likely can alter multiple ion currents. However in earlier work we have established that the antiproliferative actions of TH1177 are precisely reproduced by antagonism of TTCC using siRNA or nickel ions and not by the related channel inhibitor verapamil [3, 5]. On this basis we hypothesise that TH1177 is acting via TTCC in the current work, but this requires further verification using genetic models.

In vitro data show that both human and rat mesangial cell proliferation is dependent on T-type calcium channel activity and TTCC blockade is anti-proliferative whereas L-type calcium channel (LTCC) blockade is not [3]. The effects of TH1177 in acute thy-1 nephritis [5] and in the present work are consistent with this action. However, it is possible that in this chronic model, TH1177 is having an effect not only on cell proliferation but also on other factors involved in the progression of disease, such as the regulation of intraglomerular haemodynamics. It is likely that with the development of glomerular scleroses, the pressures within the remaining glomeruli, or indeed in the functioning capillary loops of partially sclerosed glomeruli, will increase in response to raised angiotensin II and aldosterone levels. There is evidence that TTCC blockade dilates both afferent and efferent glomerular arterioles, thereby potentially reducing the pathological rise in intraglomerular pressures that would normally occur as this disease progresses [12]. TTCC inhibition may also reduce aldosterone secretion [13] thus limiting some of the local and systemic secondary adaptive processes that contribute to ongoing glomerular damage. Effects of TH1177 on blood pressure might have contributed to the actions seen. However, we have previously demonstrated that TH1177 at these doses does not alter systemic arterial blood pressure in the WKY rat [5]. A further possible confounding effect could be the lower weight of the TH1177-treated animals (Fig. 5c). This cannot be completely ruled out, however there was no correlation between body weight and day 42 serum creatinine in the study, suggesting that lower serum creatinine values were not due to low muscle mass.

A peak in proteinuria was seen in all 3 groups at day 7, but this resolved by day 14 and there was no progressive rise in proteinuria as might have been expected with chronic glomerular injury. Previous studies of this animal model have demonstrated similar patterns of an early rise in proteinuria, peaking within the first week after antibody injection, with subsequent improvement. Most studies then show a progressive increase in proteinuria starting from 4 to 8 weeks after antibody injection (8). It is therefore possible that if this experiment were continued longer, perhaps beyond 8 weeks from injection, differences in proteinuria may have developed between the groups.

There is extensive evidence that TTCC inhibition differs from L-type calcium channel (LTCC) inhibition in both pre-clinical [14–24] and clinical studies [25–31]. In animal studies TTCC inhibition appears to offer renoprotection in a way that LTCC inhibitors do not [12, 18, 19]. The mechanisms of this difference include anti-fibrotic, antioxidant, and aldosterone lowering effects as well as favourable actions on glomerular capillary pressure [15, 17–24]. Clinical studies have examined TTCC inhibitor therapy in diabetic nephropathy, hypertension and proteinuric CKD [25–31]. In agreement with the animal data, the inhibition of TTCC in clinical studies appears to show benefits above and beyond those of LTCC inhibitors. These benefits include improved renal function, lower proteinuria and reduced serum aldosterone. Side effect profiles of TTCC appear to be benign [25–31] and problems with TTCC inhibitor mibefradil appear to have been due to cytochrome actions and not TTCC inhibition per se [32]. Further clinical study will be required to confirm safety for new TTCC inhibitor candidates.

The current study has significant limitations. The assay of creatinine by the Jaffé method is subject to errors. Accordingly, the exact size of the effects of TH1177 on serum creatinine in this model are uncertain, although such errors are unlikely to explain the differences seen. The actions of TH1177 seen in this study warrant further study as the agent appears to have benefit in reducing fibrosis even when given after the acute phase of injury has resolved, demonstrating treatment and not just prevention. In future work the use of genetic models and specific targeting with siRNA can be used to examine precise targets in more detail.

## Conclusions

The data presented here demonstrate a beneficial action of TH1177 in a rat model of chronic glomerulonephritis and glomerulosclerosis. This work provides additional support to justify further study of TH1177 and related molecules in proliferative glomerular disease. Mechanistic animal model work using highly selective ion channel inhibitors and genetic study of individual cation channel knockouts is likely to give greater insights. Further clinical study of both mixed TTCC/LTCC inhibitors and of selective TTCC inhibition will determine if these agents are an improvement on the use of LTCC inhibitors in the hypertensive patient with proliferative GN. The glomerular diseases most likely to show responses include diabetic nephropathy, IgA nephropathy and lupus nephritis.

## Supplementary information

**Additional file 1: Supplementary Fig. 1.** PAS histology of the chronic Thy1 model at days 7, 14, 28 and 42. There is progressive glomerular hypercellularity as quantified in the lower panel. **Supplementary Fig. 2.** ED1 stain for monocyte/macrophages in the chronic Thy1 model at days 7,14, 28 and 42. Scale bars are 100 μm. There is an early inflammatory increase in ED1 positive cells at day 7. This tends to reduce with few ED1 positive cells in the glomeruli at day 42 of the model. **Supplementary Fig. 3.** Ki-67 stain of the Chronic Thy1 model as an estimate of cellular proliferation with quantification in the chart at the right. Scale bars are 100 μm. The disease model shows an increase in Ki67 stain at day 28 with later resolution so that there is no difference between disease and vehicle controls by day 42. **Supplementary Fig. 4** Plot of serum creatinine against body weight at Day 42 in the therapeutic experiment on the chronic Thy1 model. There was no significant correlation seen.

## Data Availability

The datasets used and/or analysed during the current study are available from the corresponding author on reasonable request.

## Abbreviations

ANOVA: Analysis of variance
CCB: Calcium channel blocker
GN: Glomerulonephritis
IgA: Immunoglobulin A
LVA: Low voltage activated
LTCC: L-type calcium channel
MC: Mesangial cells
PAS: Periodic Acid Schiff
PMT: Picro Mallory Trichrome
siRNA: Small interfering ribonucleic acid
TTCC: T-type calcium channel
WKY: Wistar Kyoto
