# Supplementary material for: Ion-Channel modulator TH1177 reduces glomerular injury and serum creatinine in chronic mesangial proliferative disease in rats
Source: BMC Nephrol. 2020 May 19;21:187. doi: 10.1186/s12882-020-01842-5 (PMC7236127; doi:10.1186/s12882-020-01842-5)
Supplement: Supplementary file 1 — Additional file 1: Supplementary Fig. 1. PAS histology of the chronic Thy1 model at days 7, 14, 28 and 42. There is progressive glomerular hypercellularity as quantified in the lower panel. Supplementary Fig. 2. ED1 stain for monocyte/macrophages in the chronic Thy1 model at days 7,14, 28 and 42. Scale bars are 100 μm. There is an early inflammatory increase in ED1 positive cells at day 7. This tends to reduce with few ED1 positive cells in the glomeruli at day 42 of the model. Supplementary Fig. 3. Ki-67 stain of the Chronic Thy1 model as an estimate of cellular proliferation with quantification in the chart at the right. Scale bars are 100 μm. The disease model shows an increase in Ki67 stain at day 28 with later resolution so that there is no difference between disease and vehicle controls by day 42. Supplementary Fig. 4 Plot of serum creatinine against body weight at Day 42 in the therapeutic experiment on the chronic Thy1 model. There was no significant correlation seen. [file 12882_2020_1842_MOESM1_ESM.ppt]

## Slide 1
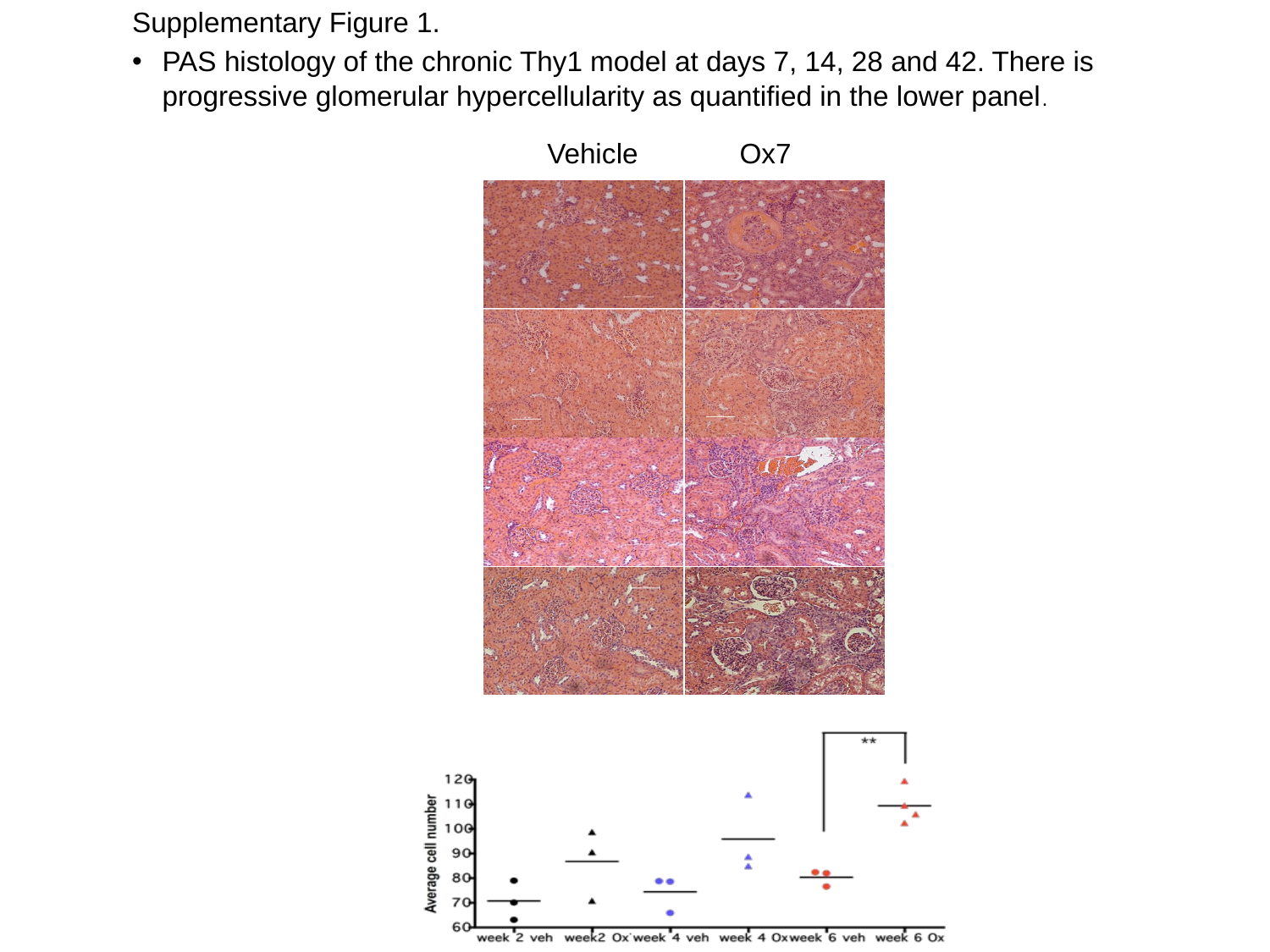

Supplementary Figure 1.
PAS histology of the chronic Thy1 model at days 7, 14, 28 and 42. There is progressive glomerular hypercellularity as quantified in the lower panel.
Vehicle Ox7

## Slide 2
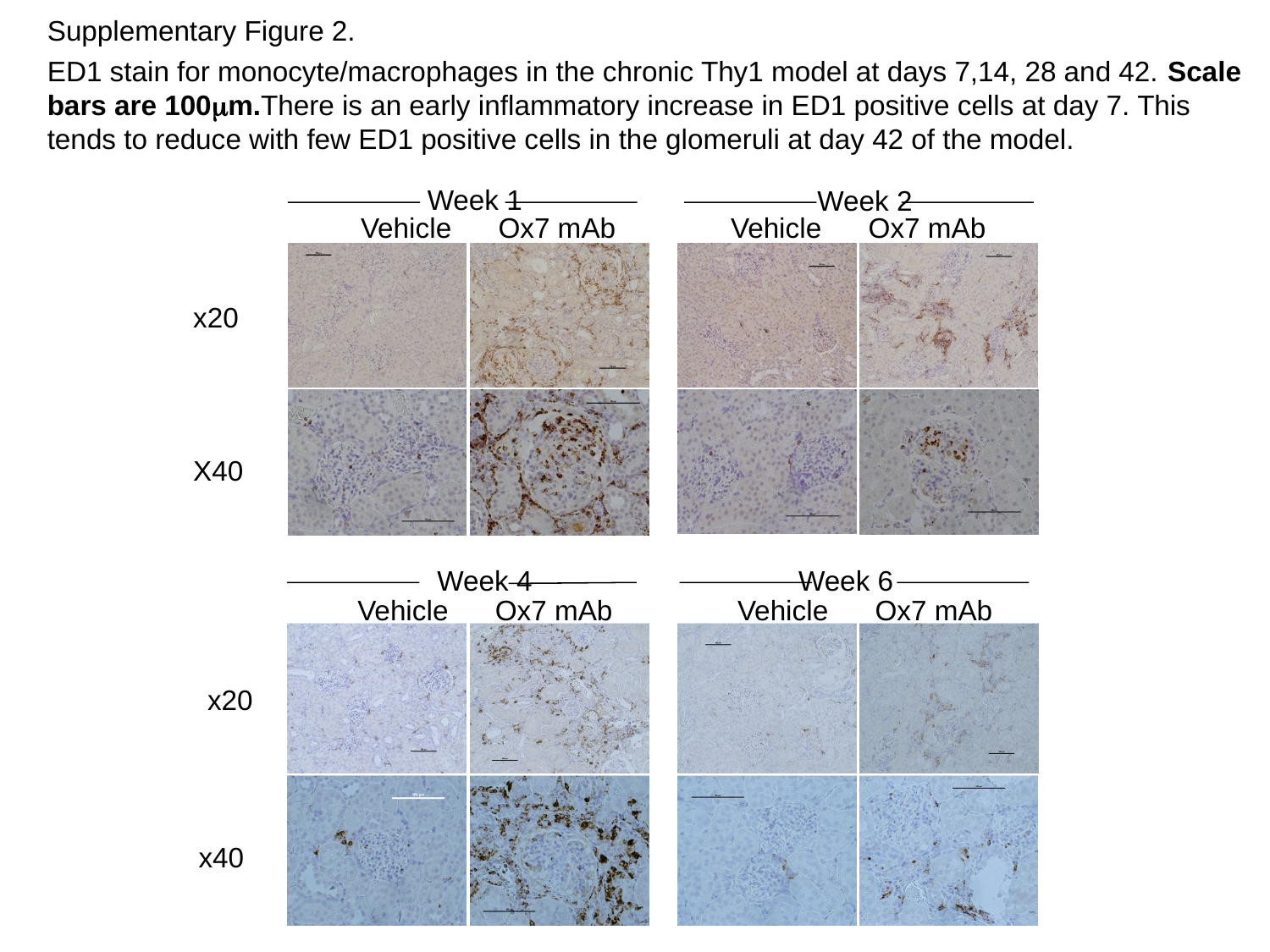

Supplementary Figure 2.
ED1 stain for monocyte/macrophages in the chronic Thy1 model at days 7,14, 28 and 42. Scale bars are 100μm.There is an early inflammatory increase in ED1 positive cells at day 7. This tends to reduce with few ED1 positive cells in the glomeruli at day 42 of the model.
Week 1
Week 2
Vehicle Ox7 mAb
Vehicle Ox7 mAb
x20
X40
Week 4
Week 6
Vehicle Ox7 mAb
Vehicle Ox7 mAb
x20
x40

## Slide 3
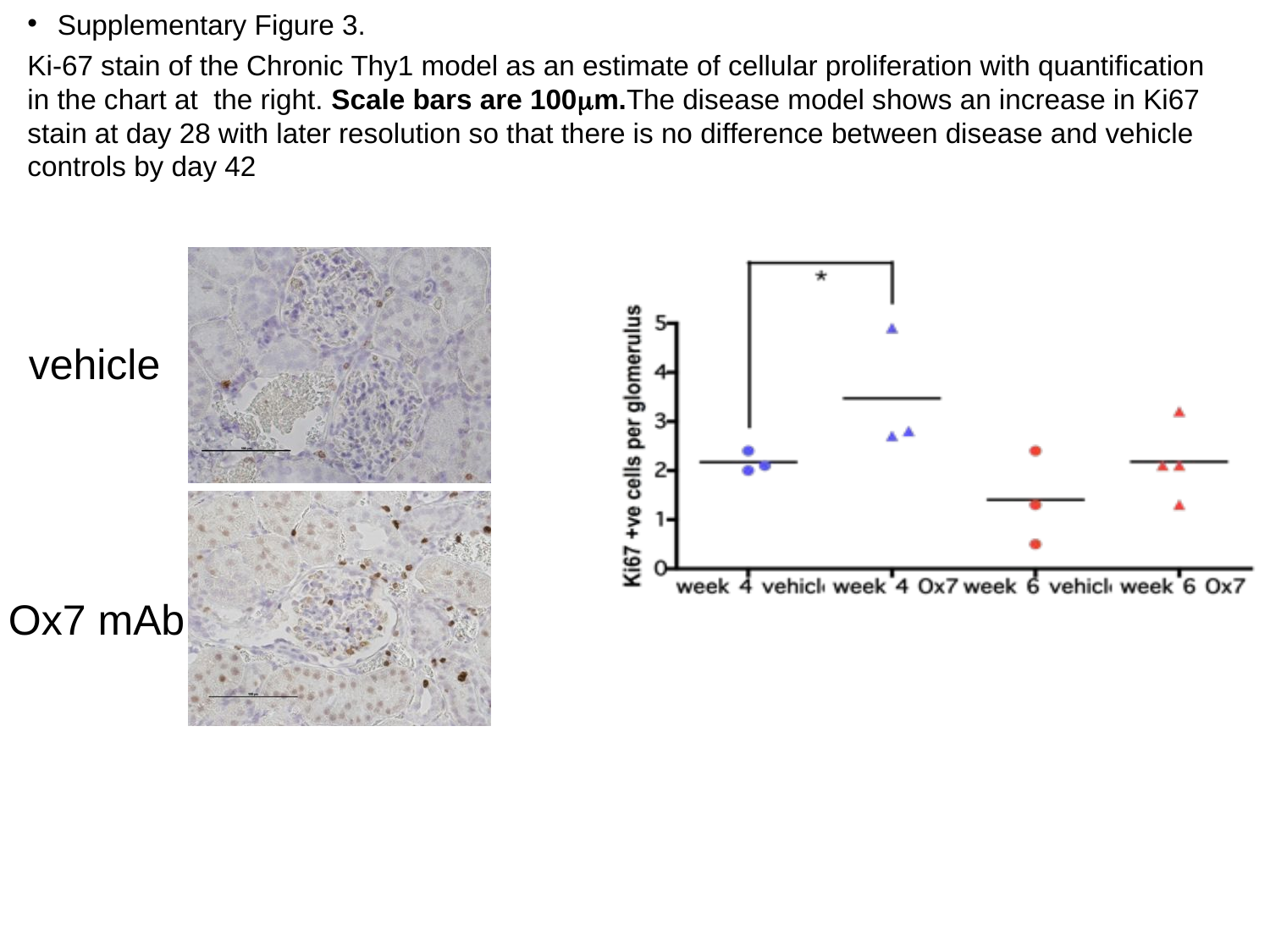

Supplementary Figure 3.
Ki-67 stain of the Chronic Thy1 model as an estimate of cellular proliferation with quantification in the chart at the right. Scale bars are 100μm.The disease model shows an increase in Ki67 stain at day 28 with later resolution so that there is no difference between disease and vehicle controls by day 42
vehicle
Ox7 mAb

## Slide 4
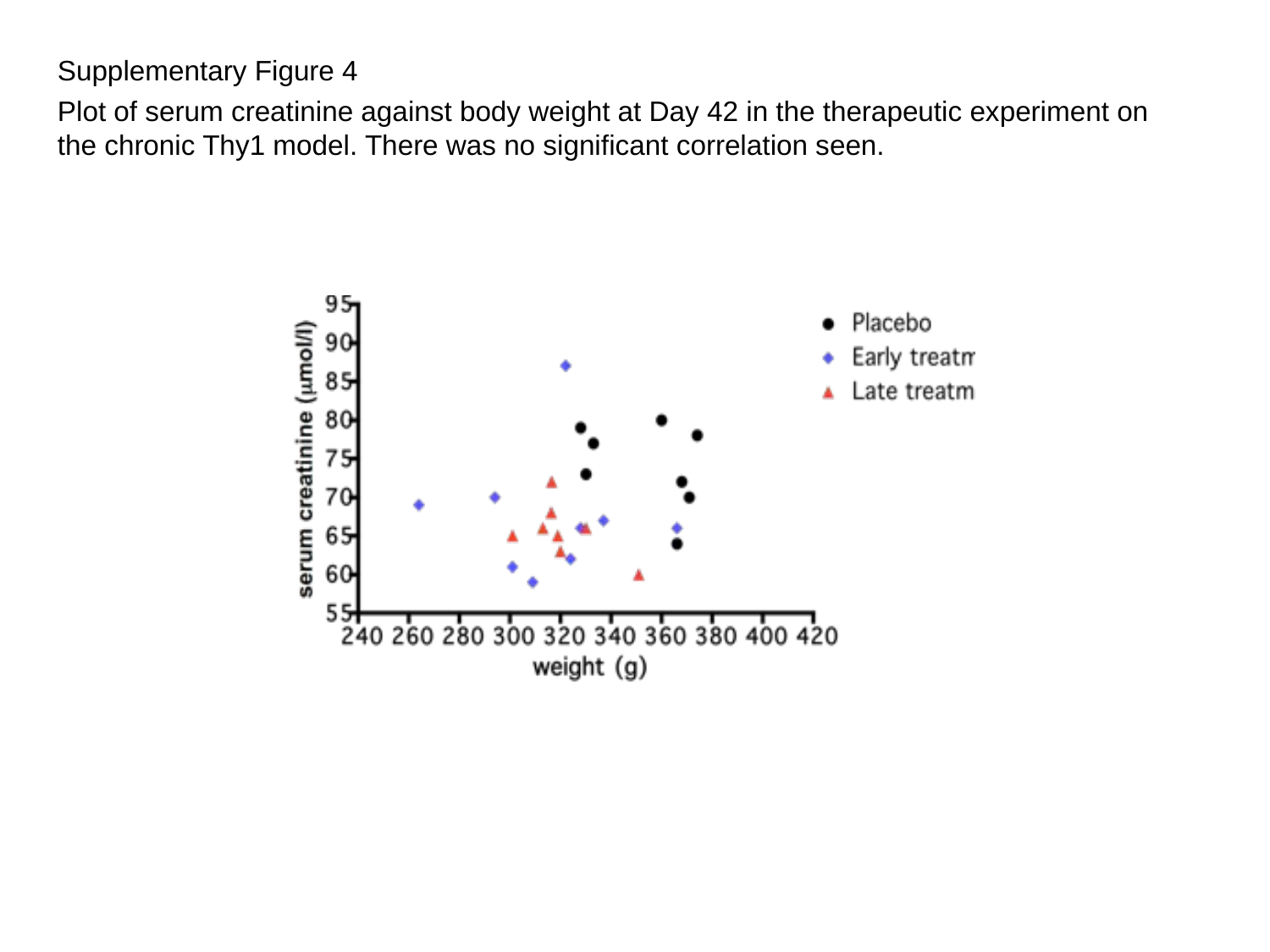

Supplementary Figure 4
Plot of serum creatinine against body weight at Day 42 in the therapeutic experiment on the chronic Thy1 model. There was no significant correlation seen.
